# An adaptive load shedding methodology for renewable integrated power systems

**DOI:** 10.1016/j.heliyon.2024.e40043

**Published:** 2024-11-01

**Authors:** Sk Fahim Abrar, Nahid-Al Masood, Mohammad Jahangir Alam

**Affiliations:** Department of Electrical and Electronic Engineering, Bangladesh University of Engineering and Technology, Dhaka, 1205, Bangladesh

**Keywords:** Load shedding, Frequency stability, ROCOF, Battery energy storage System (BESS)

## Abstract

System stability issues regarding frequency and voltage in modern power systems are growing in importance as they incorporate more and more complex components. To ensure a sustainable, pollution-free power generation, modern power systems are designed to incorporate more renewable generation sources than traditional ones. Therefore, in the event of a large-scale disruption event, conventional load-shedding strategies are unable to keep the voltage and frequency limit below the threshold value. The suggested approach takes into account this issue by rating load buses in relation to relevant frequency changes, their voltage stability, system load damping coefficients, and the introduction of green energy sources in place of fossil fuel-based ones. Battery Energy Storage Systems (BESS) are used in the proposed method to minimize load shedding amount required for conventional schemes. After determining the amount, the scheme dynamically chooses feeders as per relative weightage of the stability components (voltage, frequency) to ensure that the overall load shed amount is near to the calculated value. To verify this, the scheme is tested on IEEE 39 bus with python scripted simulation. There are four scenarios considering 250 MW, 500 MW and 1500 MW injection of PV based power generation sources with conventional generation loss of 800 MW and 1000 MW. The threshold frequency is considered 49.10 Hz. The total amount of BESS is 300 MW. For every scenario, it has been found that the methodology successfully maintains the system frequency above 49.10 Hz with a minimal amount of load shedding. Hence, the proposed methodology is able to maintain frequency stability for a modern power system with large-scale PV generation through adaptive feeder selection for load shedding.

## 1 Introduction

For keeping up with the gradually increasing demand for electricity in a dependable and sustainable manner, contemporary power systems are getting bigger and continuously incorporating advanced components [1]. The spinning reserve of the power system is dwindling as clean energy sources i.e. solar, wind, thermal, etc. are introduced. The risk of incurring frequency instability is increasing due to power grids getting larger and more complex regularly to cope with higher demand and the integration of asynchronous renewable sources [2]. To further increase the resilience of the power system in the event of a significant disruption event, voltage stability should also be considered [3]. To prevent system collapse, different load shedding schemes are implemented. Voltage or frequency factors are typically considered by classic load shedding strategies. Some frequency based Under Frequency Load Shedding scheme (UFLS) is proposed in Refs. [[4], [5], [6], [7], [8]]. These shed load in several steps based on the impact of generation loss on frequency and Rate of Change of Frequency (df/dt). Load shedding schemes utilizing voltage stability are proposed in Refs. [[9], [10], [11], [12], [13], [14]]. These kinds of schemes frequently consider various voltage stability parameters [15].

UFLS based load shedding method faces various sort of challenges on modern power systems. L. Sigrist et al. provides a comprehensive review of up to date UFLS methods for isolated power systems in Ref. [16]. The paper focuses on the generation load imbalance sensitivity on isolated grids and islands power systems. Operating the electric grid system within a specified, tolerable frequency threshold is crucial, or at the very least, preventing prolonged frequency deviations beyond this limit. As the number of spinning reserves is restricted, underfrequency operation typically poses a greater risk of potential equipment damage compared to over-frequency operation. Conventional UFLS schemes are broadly classified into experimental and optimal designs. Advanced UFLS schemes are often integrated centrally, while there are a few instances where local measurements and choices are necessary. T. Skrjanc et al. discusses about UFLS schemes using clustering method [17]. Even after clustering various UFLS into several groups, research gaps are still found which can be scope of additional work.

In [18] Larik et al. discusses the advantages and disadvantages of traditional and computational intelligence approaches while reviewing current state-of-the-art Under Voltage Load Shedding Scheme (UVLS) systems used in a variety of power utilities. Voltage collapse risk is increased by factors such natural load growth, reliance on generation located far from load centers, and shifting type of loads. In short conventional UVLS schemes fails to maintain the whereas computational intelligence methods in UVLS schemes have the potential to increase power systems' resilience and dependability. The proposed load shedding methodology combines the concept of UFLS and UVLS using computational intelligence methods to design a robust scheme that utilizes renewable integrated storage devices to minimize the load shed amount and prevent a possible blackout scenario.

Considering the schemes discussed given the occurrence of power discrepancies, most of the time these results in shedding of superfluous loads. Making an adequate load shedding plan under such conditions is a difficult task that calls for a thorough examination of the disruption event [[19], [20], [21]].

The rate of frequency change determination has been improved through the discussion and analysis of several techniques in Ref. [22]. A two-unit wide-area adaptive load shedding technology based on synchro phasors is proposed in Ref. [23]. These adaptive methods have the drawback of not accounting for the possibility that the projected disruption size may not be indicative of the real level of power loss. A result of which there can be unplanned load shedding. The impact of changing feeder load quantity is also frequently disregarded by adaptive load shedding schemes [24].

Various load forecasting methods are evaluated and addressed in Ref. [25]. This technique tries to anticipate the frequency response a few seconds in advance. So it is possible to create an adaptive load shedding strategy using such a method. Additionally, load shedding may be used by calculating the state of each relay without any intervention centrally.

In [26] a robust approach of conventional UFLS has been proposed. The proposed technique focuses on ensuring the optimum amount of load shedding so that the frequency is maintained as per the threshold value as well as prevents over shedding of loads by using predictive method. Tadej et al. proposes a methodology for fine tuning the conventional UFLS in Ref. [27]. It utilizes a set of intelligent specialized electronic devices that works with existing schemes. R. Urban et al. proposes a Rate of Change of Frequency (RoCoF) derived upgrade for existing UFLS relays that removes the problem of inflexibility in Ref. [28]. These load-shedding techniques applying a few seconds in advance frequency predictions signifies that more research work can be done.

In [29], Lazaroiu, A.C et al. discusses about the major contribution of Photovoltaic (PV) technology for worldwide transition of energy and the capacity of transforming the power sector ensuring green, effective and economic energy source. One of the limitations of utilizing PV energy resources is the lack of efficient energy storage system. This plays a vital role as solar energy is not available all the time at same level i.e. in cloudy days or nighttime. In Ref. [30], several strategies regarding battery charging for off grid solar PV system has been discussed. The comparative study presents an insightful view for enhancing efficiency, reliability and cost-effectiveness for off grid solar PV systems. In Ref. [31] Lazaroiu, G et al. provides an analysis about ensuring effective and economic energy supply to remote areas. Among different sources it was found that, battery storage system is the most feasible choice for supplying electricity to remote areas. These banks can be connected with renewable sources like solar PV system, wind turbines etc.

The lack of spinning mass associated with renewable energy sources has made it more difficult to design effective load-shedding strategies for low-inertia power systems [32,33]. Voltage Stability of Microgrids in Power Systems' prologue is covered in Ref. [34]. It offers a thorough analysis of the research on the stability of voltage in power systems having a significant proportion of inverter-based generators as part of the system's generation mix. Utility scale PV plants may include Battery Energy Storage System (BESS) to provide supplementary frequency support [35,36]. Traditional load-shedding techniques rarely consider the impact of introducing green energy sources to the electric grid.

Following the literature review and to fill these gaps, this paper aims.

• To develop a load shedding technique which will be adaptive and can be implemented in power systems with PV. The load shedding technique will concurrently take into account the voltage and frequency stability parameters. Also, BESS will be incorporated to cater additional frequency support provision. This methodology makes use of Phasor Measurement Units (PMU) voltage and frequency data.
• To analyze the effectiveness of the proposed scheme under various PV power penetration cases.
• To validate the proposed methodology, simulations are conducted on the IEEE 39 bus test system. Furthermore, performance of the proposed scheme is compared with an existing approach.

## 2 Proposed load shedding methodology

### 2.1 Step 1

The scheme monitors and records all Active Power (P_gi_) and Frequency (f_gi_) data from all generation units. In steady state operation scenario, all the generation units will have the same frequency output which is represented in equation (1),

(1) $$i.e.\;f_{g1}=f_{g2}=f_{g3}=f_{g4}=.\ldots \ldots ‥=f_{gi}=f_{sys}$$

where, f_gi_ = frequency output of i-th generator.

f_sys_ = nominal frequency in healthy state.

It is to be mentioned here that all frequency is measured in Hz and all active power is measured in Megawatts (MW). System standard frequency is 50 Hz.

### 2.2 Step 2

In this step, the amount of tolerable generation loss is verified by comparing the current and previous sample of active power from each power plant. This is represented in equations (2), (3). If equation (2) is true then it means the power imbalance is insignificant, the system will adjust it by itself. If equation (3) is true then it signifies a real generation loss event which will trigger the load shed mechanism.

(2) $$\frac{P_{Gi}\left(n-1\right)-P_{Gi}\left(n\right)}{P_{Gi}\left(n-1\right)}\times 100\%<\varepsilon$$

(3) $$\frac{P_{Gi}\left(n-1\right)-P_{Gi}\left(n\right)}{P_{Gi}\left(n-1\right)}\times 100\%>\varepsilon$$

where,

P_Gi_(n) - Present sample of real power of generation unit no i.

P_Gi_(n-1) - Previous sample of active power of generation unit no i

i – Generator Identification Number (1, 2, 3 …)

ε – Threshold value for filtering out the real generation loss event.

The value of ε controls the activation of suggested load shedding scheme. As a result, it is the system operator's concern to set the value of ε depending on the frequency control plan. Typically, it is set as 10 % or more so that real generation loss event can be filtered out from usual load variation due to weather conditions, peak to off peak variations.

### 2.3 Step 3

In steady state condition, equation (2) is true. The scheme continues to monitor and compare the active power data samples taken from different generation bus (PV). At the same time, the method calculates each load bus's Fast Voltage Stability Index (FVSI) data to determine the load shed weightage due to voltage stability. The voltage stability state of load buses can be assessed using the FVSI, which is a common tool [37,38].

FVSI of i-th bus (FVSI_i_) is calculated using equation (4):

(4) $${FVSI}_{i}=\frac{4.Z^{2}.Q_{i}}{V_{i}^{2}.X}$$

where,

Z = Line impedance in Ω.

Q_i_ = Reactive Power in MVAR.

V_i_ = i-th Bus voltage in kV.

X = Line Reactance in Ω.

Then the scheme normalizes the values of FVSI of all bus and calculates the weightage for voltage stability (A_ik_) as per equation (5):

(5) $$A_{ik}=\frac{{FVSI}_{i}}{\sum_{k=1}^{k=n}{FVSI}_{i}}$$

where,

A_ik_ = normalized value i.e. weightage for FVSI of i-th bus

n = total number of buses in the system.

After calculating all the bus FVSI values and Weightage for voltage stability (A_ik_), the scheme stores the values and continues to monitor the system with step 2.

### 2.4 Step 4

In the event of actual generation loss occurrence i.e. when (3) is true, the scheme evaluates the frequency that need to be recovered owing to load damping. The calculation for generation loss is shown in equation (6) and the frequency deviation is shown in equation (7).

(6) $$\Delta P=P_{loss}=\sum_{i=1}^{i=N}\left[P_{gi}\left(n-1\right)-P_{gi}\left(n\right)\right]$$

(7) $$\Delta f=\frac{\Delta P}{D+\frac{1}{R}}$$

where,

ΔP/P_loss_ – Generation loss amount (in MW)

N – Total number of generators contributing to the generation loss amount.

Δf – The deviation of frequency due to generation loss (in Hz)

D – Sensitivity of frequency owing to load damping (in MW/Hz)

R – Governor droop (in %)

It is to be mentioned here that the percentage of load change due to every 1 % of system frequency deviation determines load damping factor. It is typically in the range of 1 %–3 %. When there is sudden variation of load, Governor droop ensures the primary frequency response. For simulating worst possible scenario, no scope of primary frequency reserve is considered. So, the value of 1/R is considered 0 for simulation purpose.

For maintaining the system frequency within permissible threshold (f_th_ in Hz), the scheme must satisfy equation (8).

(8) $$\left(f_{sys}-\Delta f\right)\geq f_{th}$$

### 2.5 Step 5

In this step initial load shed amount (P_shed_) is calculated by equation (9).

(9) $$P_{Shed}=\left[f_{th}-\left(f_{sys}-\Delta f\right)\right]\times \left(D+\frac{1}{R}\right)$$

### 2.6 Step 6

The BESS units are connected with different PV units for energy storage purpose. They are activated in this step. Usually, the amount of this is 5%–10 % of the total generation capacity of the system. When the BESS units are connected with the grid, the load shed amount calculated in equation (9) is minimized in such a way that ensures the frequency is within the threshold value (f_TH_). The amount of reduced load amount is shown in equation (10) and the final load shed amount is depicted in (11).

(10) $$P_{reduce}=will\;be\;based\;on\;power\;loss\;and\;PV\;injection\;value$$

(11) $$P_{shed_{-}reduced}=P_{Shed}-P_{reduce}$$

### 2.7 Step 7

The rate of change of average frequency drop is used in this phase to calculate the frequency disturbance profiles caused by active power imbalance for all buses. For this, Rate of Change of Frequency (df/dt) is used. Equation (12) serves as the criteria.

(12) $$\frac{{df}_{i}}{dt}=\frac{f_{sys}-f_{i}}{t_{sys}-t_{i}}$$

where,

f_sys_ = Nominal frequency of the system before generation loss event in Hz

f_i_ = Bus frequency in Hz at time instant t_i_

t_sys_ = value of time for simulation when f_sys_ is recorded.

When calculation of df/dt is completed for all load buses, the scheme calculates the weightage for frequency stability (B_ik_) using equation (13).

(13) $$B_{ik}=\frac{\frac{{df}_{i}}{dt}}{\sum_{k=1}^{k=n}\frac{{df}_{i}}{dt}}$$

where,

B_ik_ = normalized value i.e. weightage for df/dt of i-th bus

n = total number of buses in the system.

The scheme stores the calculated values of the bus df/dt and Weightage for frequency stability (B_ik_).

### 2.8 Step 8

The scheme determines the bus weightage amount for load shed considering 50 % impact for both voltage and frequency stability using equation (14).

(14) $$W_{i}=0.5\times A_{ik}+0.5\times B_{ik}$$

where,

W_i_ = weightage for load shed for i-th bus.

### 2.9 Step 9

The load shed quantity for each bus is determined by equation (15).

(15) $$P_{shed\_i}=W_{i}\times P_{shed_{-}reduced}$$

where,

P_shed_i_ = Calculated load shed amount based on load shed weight of i-th bus.

W_i_ = load shed weight for i-th busi.

P_shed_reduced_ = Total reduced load shed amount after considering the impact of BESS.

A bus having a higher FVSI value and a high df/dt value is given priority for load shedding under the scheme. Buses are ranked according to their load shed weight (W_i_).

### 2.10 Step 10

The majority of the time, a PQ bus has several feeders. The method chooses circuit breakers for feeders so that the sum of the power flow of identified feeders is as close as possible to the load shed amount (P_shed_i_). This is depicted in equation (16).

(16) $$P_{shed_{i}}≅\sum F_{k,i}$$

where,

P_shed_i_ = Calculated load shed amount based on load shed weight of i-th bus.

F_k,i_ = load amount of k-th feeder of i-th bus in MW.

### 2.11 Step 11

In this step, if there is any difference in actual load shed amount and the calculated load shed amount for a certain bus, it is adjusted with the following bus.

The value of the discrepancy (Δ_i_) is calculated by equation (17).

(17) $$P_{shed_{i}}-\sum F_{k,i}=\left|\Delta_{i}\right|$$

The difference value (Δ_i_) is added to following bus which is depicted in equation (18).

(18) $$P_{shed\_\left(i+1\right)}=\left|\Delta_{i}\right|+P_{shed\_\left(i\right)}$$

The scheme repeats (18) until all load buses are addressed.

### 2.12 Step 12

After feeder selection is complete, the scheme compares the sum of all feeder loads with total system load shed amount calculated in (11) to ensure it is as close as possible to the value of P_shed_reduced_.

### 2.13 Step 13

**Fig. 1:**
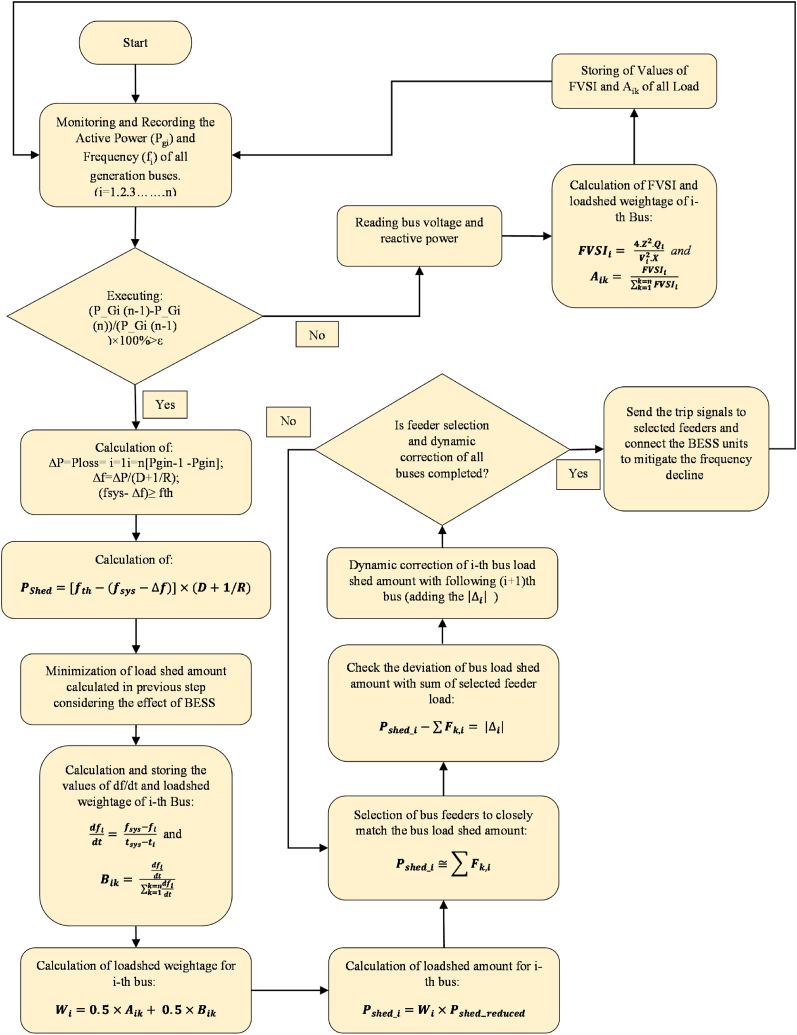
Stepwise flowchart for the devised methodology.

In order to stop the rapid frequency decline and simultaneously preserve voltage stability, fiber optic communication infrastructure is utilized by the methodology to send signals for disconnecting all specified feeder circuit breakers of all buses. After executing the script, the scheme goes back to step-1 and continues to monitor the system for any generation loss event. A flowchart of the proposed load shedding scheme is illustrated in Fig. 1.

## 3 Implementation of proposed methodology

### 3.1 Test system

**Fig. 2:**
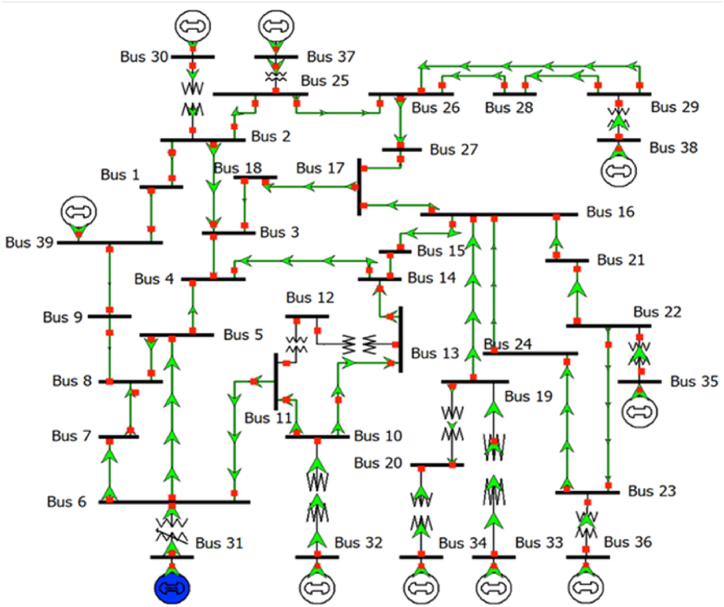
IEEE 39 bus New England Test System.

For testing purpose, IEEE 39 bus test system is a standardized power network which is shown in Fig. 2 [39]. This 39 bus test system will be used for simulation purpose of the proposed load shedding methodology. The nominal frequency is set at 50 Hz. Total load is calculated to be 6267 MW and they are distributed through 20 load buses. The software used for testing purpose is ‘DIgSILENT PowerFactory’ where Python coded scripts are written to execute the algorithm. The frequency threshold value is set at 49.10 Hz and load damping is considered to be 1.5 %.

For test purpose the system has been modified to introduce renewable resources i.e. Utility Scale PV plants. Also, it has been assumed that 20 % of this PV amount will be considered as Storage Elements (Battery Energy Storage System). It has been considered that maximum amount of PV injection will be 1500 MW and corresponding BESS amount is 300 MW. It has been assumed that the BESS will be at its maximum charged capacity when required. At the same time, power injection from conventional generating units will be replaced by amount of PV injection.

### 3.2 Simulation results and analyses

To assess how well the suggested load shedding strategy performs, four case studies are conducted. The cases are listed below.

• Case-1: 800 MW Generation Loss with PV Injection 250 MW
• Case-2: 800 MW Generation Loss with PV Injection 500 MW
• Case-3: 1000 MW Generation Loss with PV Injection 500 MW
• Case-4: 1000 MW Generation Loss with PV Injection 1500 MW

The results of all simulation cases are mentioned below.

#### 3.2.1 Case-1: 800 MW generation loss with PV injection 250 MW

In this case a sudden disconnection of 800 MW generation is considered (i.e. ΔP = 800 MW). The threshold frequency is set at 49.10 Hz. Total available load is about 6267 MW and the value for load damping factor is assumed 1.5 %. As per these values, the frequency sensitivity (D) is calculated as (6267 × 0.015)/(50 × 0.01) MW/Hz = 188.00 MW/Hz. From equation (7), the value of frequency deviation (Δf) is = 800/188.00 Hz = 4.25 Hz. Then from equation (9) the initial load shed size P_shed_ is = [49.10 - (50–4.25)] × 188.00 MW ≈ 629.8 MW. Later, the load shed value is reduced to 235 MW by the scheme based on the value of generation loss and PV injection. For this case the generation loss is considered 800 MW and PV injection is 250 MW.

Then the proposed scheme calculates the FVSI and df/dt values of all load bus and ranks them based on the weightage for load shed. All the calculations are shown on Table 1.

**Table 1:** Load Bus Data for Case 1.

Sl. No.	Bus Number	FVSI Value	df/dt Value	Loadshed weight	Bus Rank	Calculated Loadshed Amount (MW)
1	Bus_28	0.01508517	−0.01189873	0.0229972	19	5
2	Bus_29	0.02446216	−0.01121979	0.0268385	17	6
3	Bus_18	0.01798516	−0.01802119	0.0326152	15	8
4	Bus_25	0.02619467	−0.01108944	0.0281559	16	7
5	Bus_26	0.0096458	−0.01408122	0.0232091	18	5
6	Bus_27	0.04499852	−0.01687125	0.0447445	11	11
7	Bus_16	0.01940876	−0.02439278	0.041149	12	10
8	Bus_21	0.07158801	−0.03156536	0.0773269	4	18
9	Bus_22	0.07084238	−0.03958498	0.0867852	1	20
10	Bus_23	0.05066428	−0.03719812	0.0730218	6	17
11	Bus_24	0.04151349	−0.02612382	0.0547143	8	13
12	Bus_14	0.06193118	−0.01685715	0.05464	9	13
13	Bus_15	0.09571123	−0.02213958	0.0775631	3	18
14	Bus_20	0.06430172	−0.02148285	0.0605873	7	14
15	Bus_7	0.05175012	−0.01390085	0.0471014	10	11
16	Bus_8	0.10948849	−0.01349269	0.0758371	5	18
17	Bus_12	0.03242101	−0.01526431	0.0380234	13	9
18	Bus_3	0.00178257	−0.01447111	0.0201604	20	5
19	Bus_4	0.11525705	−0.01538558	0.0803193	2	19
20	Bus_39	0.05688958	−0.00362954	0.0342105	14	8
**Total Load Shed**						**235**

Table 1 shows the initially calculated values by the scheme. In the consequent step the scheme selects the bus feeders to ensure the entire load shed amount (238 MW) is close to the calculated load shed amount (235 MW). The actual load shedding scenario is depicted in Table 2.

**Table 2:** Feeder Data for Case 1.

Sl. No.	Bus Number	Calculated Loadshed Amount (MW)	Actual Loadshed (MW)	Dynamic Correction Value (MW)	Feeder Number	Feeder Load
1	Bus_28	5	0	5	N/A	0
2	Bus_29	6	0	11	N/A	0
3	Bus_18	8	23	−4	Feeder 3	23
4	Bus_25	7	0	3	N/A	0
5	Bus_26	5	10	−2	Feeder 4	10
6	Bus_27	11	0	9	N/A	0
7	Bus_16	10	20	−1	Feeder 5	20
8	Bus_21	18	20	−3	Feeder 5	20
9	Bus_22	20	20	−3	Feeder 5	20
10	Bus_23	17	15	−1	Feeder 5	15
11	Bus_24	13	15	−3	Feeder 6	15
12	Bus_14	13	0	10	N/A	0
13	Bus_15	18	30	−2	Feeder 5	30
14	Bus_20	14	0	12	N/A	0
15	Bus_7	11	20	3	Feeder 5	20
16	Bus_8	18	20	1	Feeder 5	20
17	Bus_12	9	10	0	Feeder 5	10
18	Bus_3	5	0	5	N/A	0
19	Bus_4	19	20	4	Feeder 6	20
20	Bus_39	8	15	−3	Feeder 7	15
**Total**		**235**	**238**	**−3**		**238**

From Table 2 the value of actual load shed amount is 238 MW. At the same time there is addition of BESS units amounting 300 MW distributed throughout the system to assist frequency arresting process. So, a total of 535 MW (235 MW load shed of feeders and 300 MW addition of BESS) switching events occur in the generation loss scenario.

**Fig. 3:**
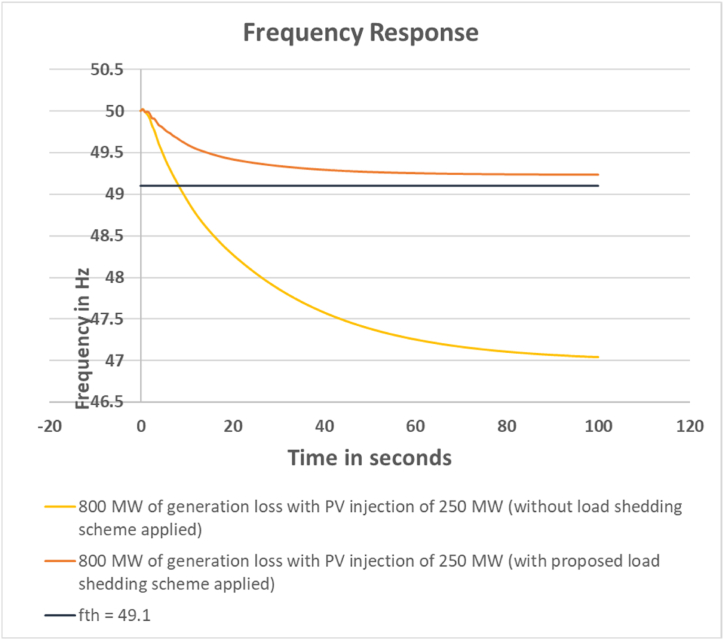
Frequency response for 800 MW generation loss with PV injection 250 MW.

The frequency response of the system with and without applying the load shedding scheme is depicted in Fig. 3.

It can be seen that; the scheme has been successful in maintaining the power network frequency within the threshold limit.

#### 3.2.2 Case-2: 800 MW generation loss with PV injection 500 MW

For this case, PV injection amount has been increased to 500 MW and generation loss is 800 MW. The available generation amount is similar to previous case with just exception of 250 MW additional PV generation in place of conventional sources. All the relevant calculation for this case is depicted in Table 3.

**Table 3:** Load Bus Data for Case 2.

Sl. No.	Bus Number	FVSI Value	df/dt Value	Loadshed weight	Bus Rank	Calculated Loadshed Amount (MW)
1	Bus_28	0.01508517	−0.01189873	0.0229972	19	5
2	Bus_29	0.02446216	−0.01121979	0.0268385	17	6
3	Bus_18	0.01798516	−0.01802119	0.0326152	15	8
4	Bus_25	0.02619467	−0.01108944	0.0281559	16	7
5	Bus_26	0.0096458	−0.01408122	0.0232091	18	5
6	Bus_27	0.04499852	−0.01687125	0.0447445	11	11
7	Bus_16	0.01940876	−0.02439278	0.041149	12	10
8	Bus_21	0.07158801	−0.03156536	0.0773269	4	18
9	Bus_22	0.07084238	−0.03958498	0.0867852	1	20
10	Bus_23	0.05066428	−0.03719812	0.0730218	6	17
11	Bus_24	0.04151349	−0.02612382	0.0547143	8	13
12	Bus_14	0.06193118	−0.01685715	0.05464	9	13
13	Bus_15	0.09571123	−0.02213958	0.0775631	3	18
14	Bus_20	0.06430172	−0.02148285	0.0605873	7	14
15	Bus_7	0.05175012	−0.01390085	0.0471014	10	11
16	Bus_8	0.10948849	−0.01349269	0.0758371	5	18
17	Bus_12	0.03242101	−0.01526431	0.0380234	13	9
18	Bus_3	0.00178257	−0.01447111	0.0201604	20	5
19	Bus_4	0.11525705	−0.01538558	0.0803193	2	19
20	Bus_39	0.05688958	−0.00362954	0.0342105	14	8
**Total Load Shed**						**235**

From Table 3 it is seen that the calculations are pretty similar to previous with some changes in stability margins that occurred due to increase in injection of PV generation. The corresponding actual load shed scenario is listed in Table 4.

**Table 4:** Feeder Data for Case 2.

Sl. No.	Bus Number	Calculated Loadshed Amount (MW)	Actual Loadshed (MW)	Dynamic Correction Value (MW)	Feeder Number	Feeder Load
1	Bus_28	5	0	5	N/A	0
2	Bus_29	6	0	11	N/A	0
3	Bus_18	8	23	−4	Feeder 3	23
4	Bus_25	7	0	3	N/A	0
5	Bus_26	5	10	−2	Feeder 4	10
6	Bus_27	11	0	9	N/A	0
7	Bus_16	10	20	−1	Feeder 5	20
8	Bus_21	18	20	−3	Feeder 5	20
9	Bus_22	20	20	−3	Feeder 5	20
10	Bus_23	17	15	−1	Feeder 5	15
11	Bus_24	13	15	−3	Feeder 6	15
12	Bus_14	13	0	10	N/A	0
13	Bus_15	18	30	−2	Feeder 5	30
14	Bus_20	14	0	12	N/A	0
15	Bus_7	11	20	3	Feeder 5	20
16	Bus_8	18	20	1	Feeder 5	20
17	Bus_12	9	10	0	Feeder 5	10
18	Bus_3	5	0	5	N/A	0
19	Bus_4	19	20	4	Feeder 6	20
20	Bus_39	8	15	−3	Feeder 7	15
**Total**		**235**	**238**	**−3**		**238**

**Fig. 4:**
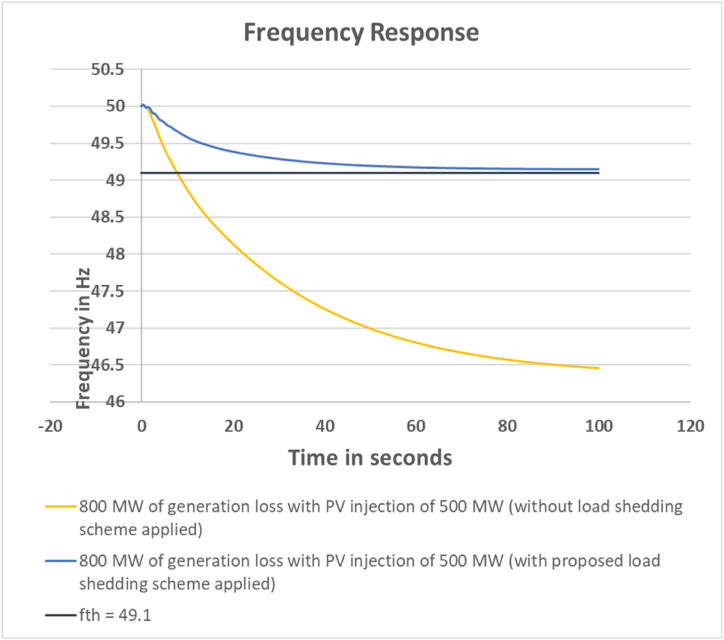
Frequency response for 800 MW generation loss with PV injection 500 MW.

For this case the devised scheme has successfully kept the grid frequency within 49.10 Hz which is illustrated in Fig. 4.

#### 3.2.3 Case-3: 1000 MW generation loss with PV injection 500 MW

For this scenario, the generation loss increased to 1000 MW and PV injection is 500 MW. The loss of 1000 MW results in higher amount of load shed to maintain the system frequency within the threshold limit. The parameter calculations are shown in Table 5, Table 6.

**Table 5:** Load Bus Data for Case 3.

Sl. No.	Bus Number	FVSI Value	df/dt Value	Loadshed weight	Bus Rank	Calculated Loadshed Amount (MW)
1	Bus_28	0.01505528	−0.01189873	0.0229009	19	9
2	Bus_29	0.02442893	−0.01121979	0.0266901	17	11
3	Bus_18	0.01816902	−0.01802119	0.032611	15	13
4	Bus_25	0.02611998	−0.01108944	0.0279772	16	11
5	Bus_26	0.00960866	−0.01408122	0.0231384	18	9
6	Bus_27	0.04490956	−0.01687125	0.0444573	11	18
7	Bus_16	0.019616	−0.02439278	0.0411489	12	16
8	Bus_21	0.07253703	−0.03156536	0.0774194	4	31
9	Bus_22	0.07190136	−0.03958498	0.0869372	1	35
10	Bus_23	0.05137466	−0.03719812	0.0731069	6	29
11	Bus_24	0.04201241	−0.02612382	0.0547421	9	22
12	Bus_14	0.06282294	−0.01685715	0.0547557	8	22
13	Bus_15	0.09706878	−0.02213958	0.0777316	3	31
14	Bus_20	0.06444147	−0.02148285	0.0603114	7	24
15	Bus_7	0.05250906	−0.01390085	0.0472051	10	19
16	Bus_8	0.11112759	−0.01349269	0.0760733	5	30
17	Bus_12	0.0328055	−0.01526431	0.0380425	13	15
18	Bus_3	0.00180756	−0.01447111	0.0201634	20	8
19	Bus_4	0.11721203	−0.01538558	0.0806835	2	32
20	Bus_39	0.05688958	−0.00362954	0.0339041	14	14
**Total Load Shed**						**399**

**Table 6:** Feeder Data for Case 3.

Sl. No.	Bus Number	Calculated Loadshed Amount (MW)	Actual Loadshed (MW)	Dynamic Correction Value (MW)	Feeder Number	Feeder Load
1	Bus_28	9	10	−1	Feeder 7	10
2	Bus_29	11	0	10	N/A	0
3	Bus_18	13	23	0	Feeder 3	23
4	Bus_25	11	0	11	N/A	0
5	Bus_26	9	20	0	Feeder 3	20
6	Bus_27	18	21	−3	Feeder 5	21
7	Bus_16	16	0	13	N/A	0
8	Bus_21	31	0	44	N/A	0
9	Bus_22	35	70	9	Feeder 1	70
10	Bus_23	29	30	8	Feeder 2	30
11	Bus_24	22	30	0	Feeder 4	30
12	Bus_14	22	25	−3	Feeder 6	25
13	Bus_15	31	30	−2	Feeder 5	30
14	Bus_20	24	20	2	Feeder 6	20
15	Bus_7	19	20	1	Feeder 5	20
16	Bus_8	30	30	1	Feeder 4	30
17	Bus_12	15	15	1	Feeder 2	15
18	Bus_3	8	12	−3	Feeder 6	12
19	Bus_4	32	30	−1	Feeder 5	30
20	Bus_39	14	15	−2	Feeder 7	15
**Total**		**399**	**401**	**−2**		**401**

**Fig. 5:**
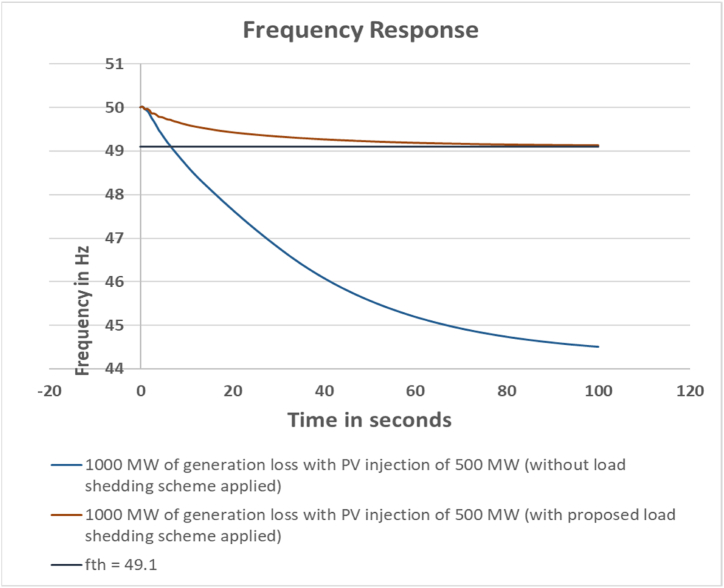
Frequency response for 1000 MW generation loss with PV injection 500 MW.

The calculated load shed amount has increased to 399 MW. Corresponding actual scenario in the system shows that 400 MW of feeders are disconnected to meet the frequency criteria. The frequency response is shown in Fig. 5.

For this case the devised scheme has successfully kept the grid frequency within 49.10 Hz.

#### 3.2.4 Case-4: 1000 MW generation loss with PV injection 1500 MW

For the final case it has been considered that the system will face a 1000 MW generation loss with full 1500 MW injection of PV power. In this scenario 3 of the conventional generation units in the test system are replaced with PV power injection. The simulation data are listed in Table 7, Table 8.

**Table 7:** Load Bus Data for Case 4.

Sl. No.	Bus Number	FVSI Value	df/dt Value	Loadshed weight	Bus Rank	Calculated Loadshed Amount (MW)
1	Bus_28	0.01527624	−0.01189873	0.0225014	19	15
2	Bus_29	0.02472145	−0.01121979	0.0260108	17	17
3	Bus_18	0.01937829	−0.01802119	0.0325723	14	21
4	Bus_25	0.02651237	−0.01108944	0.0272885	16	18
5	Bus_26	0.00996134	−0.01408122	0.0229831	18	15
6	Bus_27	0.04728148	−0.01687125	0.0440714	11	29
7	Bus_16	0.02091815	−0.02439278	0.0411055	12	27
8	Bus_21	0.07671314	−0.03156536	0.0769585	4	50
9	Bus_22	0.07531771	−0.03958498	0.0861401	1	56
10	Bus_23	0.05325788	−0.03719812	0.0722749	6	47
11	Bus_24	0.0446766	−0.02612382	0.0545906	9	36
12	Bus_14	0.07006856	−0.01685715	0.0560634	8	36
13	Bus_15	0.1057537	−0.02213958	0.0785712	3	51
14	Bus_20	0.07153543	−0.02148285	0.0614936	7	40
15	Bus_7	0.05677526	−0.01390085	0.0474562	10	31
16	Bus_8	0.12009415	−0.01349269	0.0765753	5	50
17	Bus_12	0.03697075	−0.01526431	0.0389049	13	25
18	Bus_3	0.00193205	−0.01447111	0.0201615	20	13
19	Bus_4	0.12892685	−0.01538558	0.0822749	2	54
20	Bus_39	0.05688958	−0.00362954	0.0320019	15	21
**Total Load Shed**						**652**

**Table 8:** Feeder Data for Case 4.

Sl. No.	Bus Number	Calculated Loadshed Amount (MW)	Actual Loadshed (MW)	Dynamic Correction Value (MW)	Feeder Number	Feeder Load
1	Bus_28	15	16	−1	Feeder 6	16
2	Bus_29	17	0	16	N/A	0
3	Bus_18	21	30	7	Feeder 2	30
4	Bus_25	18	24	1	Feeder 5	24
5	Bus_26	15	15	1	Feeder 5	15
6	Bus_27	29	30	0	Feeder 2	30
7	Bus_16	27	29	−2	Feeder 7	29
8	Bus_21	50	50	−2	Feeder 3	50
9	Bus_22	56	50	4	Feeder 3	50
10	Bus_23	47	50	1	Feeder 3	50
11	Bus_24	36	30	7	Feeder 4	30
12	Bus_14	36	30	13	Feeder 4	30
13	Bus_15	51	45	19	Feeder 4	45
14	Bus_20	40	0	59	N/A	0
15	Bus_7	31	0	90	N/A	0
16	Bus_8	50	130	10	Feeder 1 + Feeder 4	130
17	Bus_12	25	22	13	Feeder 2 + Feeder 6	22
18	Bus_3	13	12	14	Feeder 6	12
19	Bus_4	54	50	18	Feeder 4	50
20	Bus_39	21	25	14	Feeder 8	25
**Total**		**652**	**638**	**14**		**638**

**Fig. 6:**
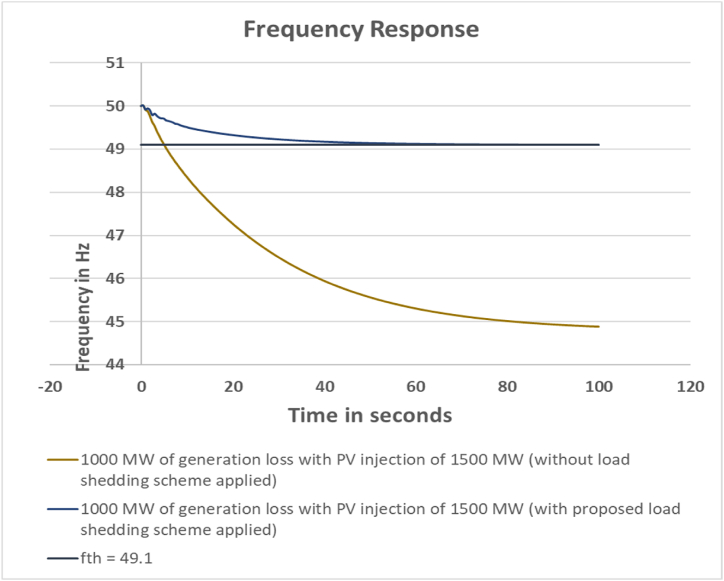
Frequency response for 1000 MW generation loss with PV injection 1500 MW.

From Table 7, Table 8, total calculated load shed amount is 652 MW whereas total actual load shed amount is 638 MW based on test system feeder availability. Just like previous cases the BESS addition amount is still 300 MW. The system frequency response is shown in Fig. 6.

From the frequency excursion curve, it can be said that the devised load shedding scheme has successfully maintained the frequency threshold limit even in the most extreme contingency condition.

**Fig. 7:**
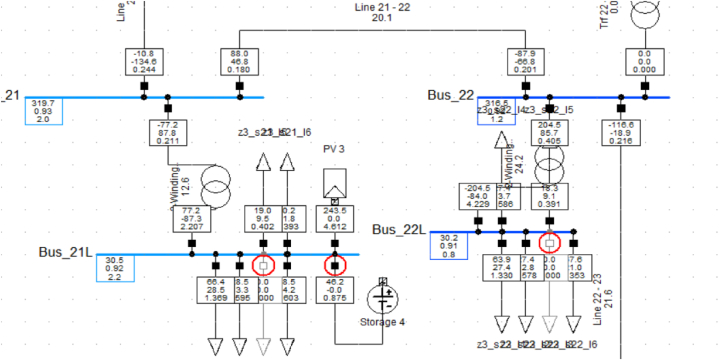
A portion of switching events occurring in the test system due to application of the load shedding scheme.

A portion of the test system after application of the scheme is shown in Fig. 7. It can be observed that red circled breakers are operated. The data transfer rate of the PMUs employed in the simulations is 25 readings per second for a 50 Hz power system which is set as IEEE Synchrophasor, C37.118 [40].

### 3.3 Critical parameters

To identify the crucial elements of the suggested load shedding technique, a sensitivity analysis is carried out. In order to evaluate the effectiveness of the suggested technique, various parameters are step-wise modified. The following are the main parameters that play a vital role on the suggested scheme.

a. Generation loss amount.
b. Frequency sensitivity due to Load Damping.
c. Rate of Change of Frequency (df/dt).
d. Systems threshold frequency set by the Network Operator.
e. Governor Droop.
f. Fast Voltage Stability Index (FVSI).
g. Amount of Renewable Energy Sources.
h. Amount of BESS injected

### 3.4 Wide area measurement systems (WAMS) limitations and time delays

**Fig. 8:**
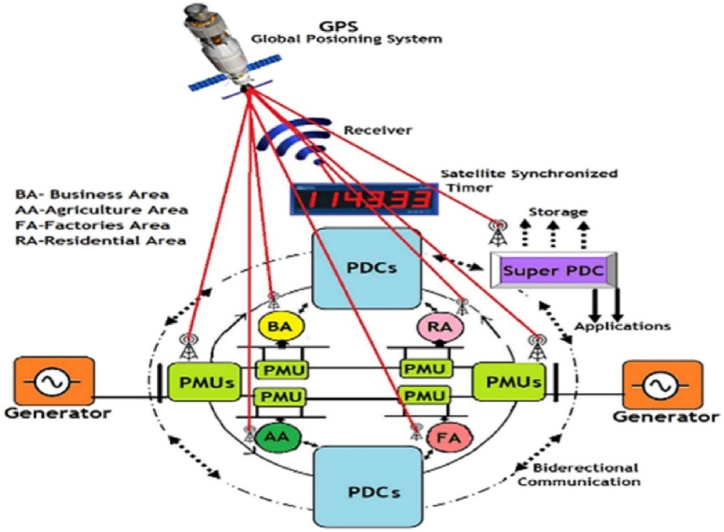
A typical WAMS system and its components.

By employing the wide-area monitoring system (WAMS) as mentioned in Ref. [41], grid operators can improve various aspects of an electrical grid. Fig. 8 shows a general setup of a WAMS environment. To obtain precise time stamping across the power grid structure, the system makes use of a Global Positioning System (GPS) in conjunction with power phasor data concentrators (PDCs) and Phasor Measurement Units (PMUs) that are systematically positioned. One of the primary challenges when utilizing WAMS is assessing real-time transient stability quickly and precisely through measurements. Major drawbacks for WAMS-based load shedding schemes include the lack of an accurate load model, insufficient training data for machine learning-based applications, longer communication times between PMUs and central control centers, costly communication structures, problems with data quality and cyber security, etc. System operators have more work ahead of them due to a combination of reduced inertia, more sensitive voltage profiles, and increased renewable energy generation. This has caused power engineers to concentrate on developing novel detection and protection systems that make use of PMU and WAMS.

Time delays for various activities are involved in case of real time centralized load shedding decision making that relies on PMUs. Usually, the delays consists of parameter reading, round-trip communication latency, the operation of the load circuit breakers, and the calculation time for the suggested algorithm. Table 9 provides a list of conventional delays due to various tasks based on WAMS. These are measurement filtering within PMU (up to 100 ms), PMU unit processing (up to 30 ms) and application input (up to 5 ms). In normal case, PDC processing and alignment (between 2 ms and 2 s) and communication system buffering and error corrections (between 0.05 ms and 8 s) do not create any issue. It can be considered that the sum of time delay for collecting measurements towards the control center will be roughly T_tot_ = 300 ms by adding the above values if the physical distances of the generation units under consideration are within 1000 km margin (6 ms) [42].

**Table 9:** List of Delays for WAMS.

Reason of Delay	Lowest Time Delay (ms)
Time required for reading parameters/Measurements	60
Round-trip communication medium (Fibre optic based) latency	150
Load circuit breaker switching	30
Computational time for the proposed technique	60
Sum of delay	300

### 3.5 Issues with distributed photovoltaic generation

The suggested methodology is tested in the IEEE 39 bus test system with Utility Scale Photovoltaic (PV) generation in place. The feeder selection procedure is python coded script which runs in DigSilent PowerFactory environment. Different element type is modelled using different suffices. As such even if the PV units are connected as distributed generation the script can easily identify the feeders of load elements that need to be shed.

### 3.6 Sensitivity analysis: variation of feeder load shed time

**Fig. 9:**
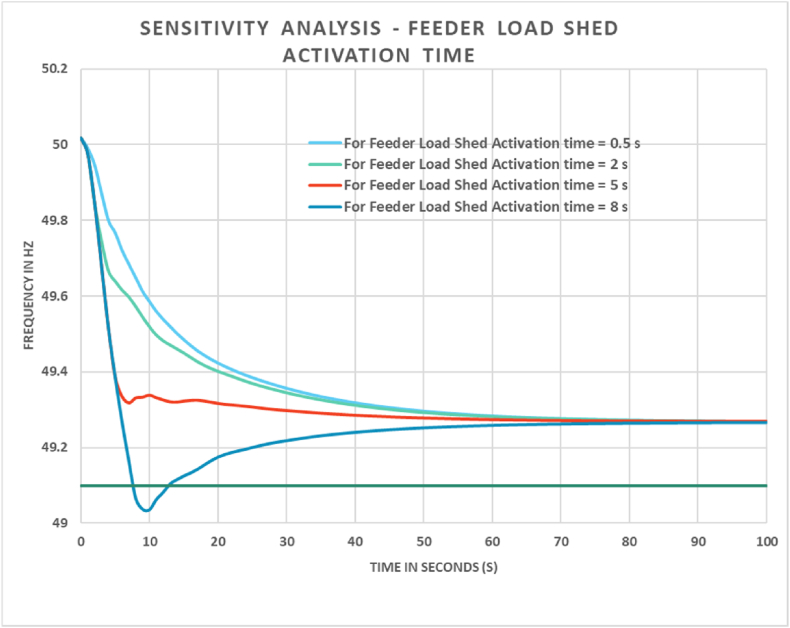
Effect of variation of feeder load shed activation time on frequency response.

As mentioned in Ref. [7], optimal UFLS activation time remains within 1 s of occurrence of generation loss event. To filter out unnecessary load shedding, the scheme has provision for filtering out the real generation loss events from normal frequency deviation of grid. For simulation cases, the feeder load shed time was set to 0.50 s after loss of generation. Furthermore, a sensitivity analysis of this load shed time was performed for Case 4 to compare the results which is shown in Fig. 9.

From Fig. 9 it can be observed that, the rate of frequency arrest deteriorates with increase of load shed time. For feeder load shed activation time greater than 8 s the scheme fails to satisfy the frequency limit of the network. Additional modification can be done to rectify this. But in practical scenario the load shed schemes are activated well before this timeframe.

### 3.7 Sensitivity analysis: variation of voltage and frequency stability index weightage

**Fig. 10:**
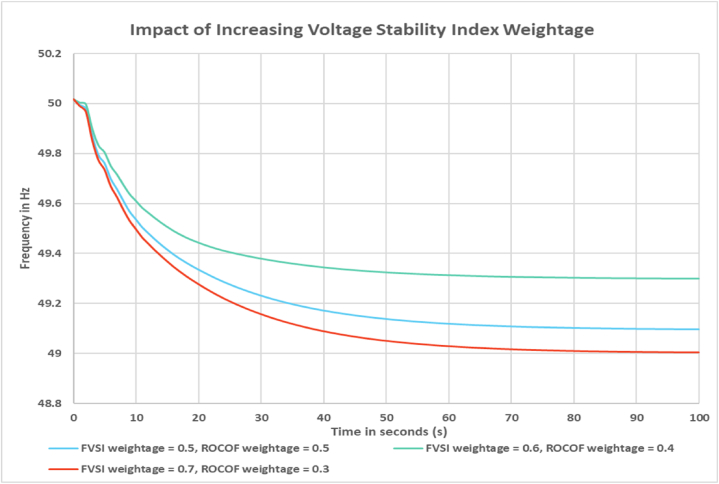
Impact of increased Voltage Stability Index weightage on frequency response.

**Fig. 11:**
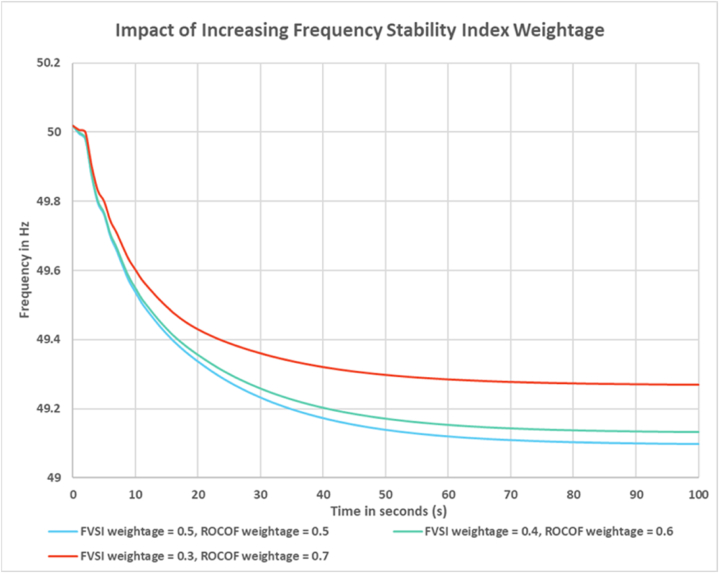
Impact of increased Voltage Stability Index weightage on frequency response.

For the simulation purpose, the weightage for voltage and frequency stability index was set to 50 %. In this section this weightage will be varied to observe how the output frequency changes. From Fig. 10 it can be observed that when the weightage for FVSI is increased to 0.6, the frequency response improves. Which implies a lower amount of load shed than previous scenario. When the weightage is increased further to 0.7 the frequency response deteriorates. From Fig. 11 it can be observed that when the weightage for ROCOF is increased to 0.6, the frequency response improves. Which again means lower amount of load shed can be done to maintain frequency limit. When the weightage is increased further to 0.7 the frequency response deteriorates. From the comparison, it can be said that the ROCOF weightage may be increased to improve the frequency response of the system. In other words, it signifies that the system is more susceptible to frequency stability compared to voltage stability of the load buses. The weightage of the indices can be fixed by the system operator based on the need and grid code.

### 3.8 Derivation of fast Voltage Stability Index (FVSI)

For a two-bus power system, bus-1 being the sending end and bus-2 being the receiving end. Line current I is given by equation (19).

(19) $$I=\frac{V_{1}∠\delta_{1}-V_{2}∠\delta_{2}}{R+jX}$$

where,

V_1_ = Sending end voltage magnitude.

V_2_ = Receiving end voltage magnitude

δ_1_ = Sending end voltage angle

δ_2_ = Receiving end voltage angle.

R = Resistance of the line connecting sending and receiving end.

X = Reactance of the line connecting sending and receiving end.

If following are assumed,

P_1_ = Sending end real power.

P_2_ = Receiving end real power.

Q_1_ = Sending end reactive power.

Q_2_ = Receiving end reactive power.

The apparent power at receiving end (S_2_) is shown in equation (20).

(20) $$S_{2}=V_{2}I^{*}$$

Re-arranging (20) equation (21) can be obtained,

(21) $$I={\left(\frac{S_{2}}{V_{2}}\right)}^{*}=\frac{P_{2}-{jQ}_{2}}{V_{2}∠-\delta_{2}}$$

From (19) and (21),

(22) $$\frac{P_{2}-{jQ}_{2}}{V_{2}∠-\delta_{2}}=\frac{V_{1}∠\delta_{1}-V_{2}∠\delta_{2}}{R+jX}$$

Assuming δ = δ_1_ - δ_2_ in equation (22) yields equation (23),

(23) $$V_{1}V_{2}∠\delta -V_{2}^{2}=\left(R+jX\right)\left(P_{2}-jQ_{2}\right)$$

Separating the complex value in (23), real part is given by equation (24) and imaginary part is given by equation (25)

(24) $$V_{1}V_{2}\;\cos\;\delta -V_{2}^{2}=RP_{2}+XQ_{2}$$

(25) $$V_{1}V_{2}\;\sin\;\delta =XP_{2}-RQ_{2}$$

Replacing P_2_ from equation (25) into equation (24), the quadratic expression of V_2_ is obtained which is depicted in equation (26).

(26) $$V_{2}^{2}+V_{1}V_{2}\left(\frac{R\;\sin\;\delta }{X}-\cos\;\delta \right)+Q_{2}\left(\frac{R^{2}}{X}+X\right)=0$$

The roots of V_2_ are given in equation (27).

(27) $$V_{2}=\frac{-V_{1}\left(\frac{R\;\sin\;\delta }{X}-\cos\;\delta \right)\pm \sqrt{V_{1}^{2}{\left(\frac{R\;\sin\;\delta }{X}-\cos\;\delta \right)}^{2}-4Q_{2}\left(\frac{R^{2}}{X}+X\right)}}{2}$$

For a real root for V_2_, equation (28) must be satisfied.

(28) $$V_{1}^{2}{\left(\frac{R\;\sin\;\delta }{X}-\cos\;\delta \right)}^{2}-4Q_{2}\left(\frac{R^{2}}{X}+X\right)\geq 0$$

Since, δ is normally small, it can be considered Rsinδ ≈ 0 and Xcosδ ≈ X, therefore eq (28) can be rewritten as per equation (29).

(29) $$\frac{4Q_{2}\left(\frac{R^{2}}{X}+X\right)}{V_{1}^{2}}\leq 1$$

equation (29) can be rewritten as equation (30).

(30) $$\frac{4Q_{2}\left(R^{2}+X^{2}\right)}{V_{1}^{2}X}\leq 1$$

Replacing (R_2_+X_2_) = Z_2_, equation (30) can be rewritten as equation (31).

(31) $$\frac{4Q_{2}Z^{2}}{V_{1}^{2}X}\leq 1$$

Setting V_1_ = V_i_ in equation (31) the FVSI for bus no i can be expressed as equation (32).

(32) $${FVSI}_{i}=\frac{4Q_{i}Z^{2}}{V_{i}^{2}X}$$

### 3.9 Load shedding methodology in case of system splits

**Fig. 12:**
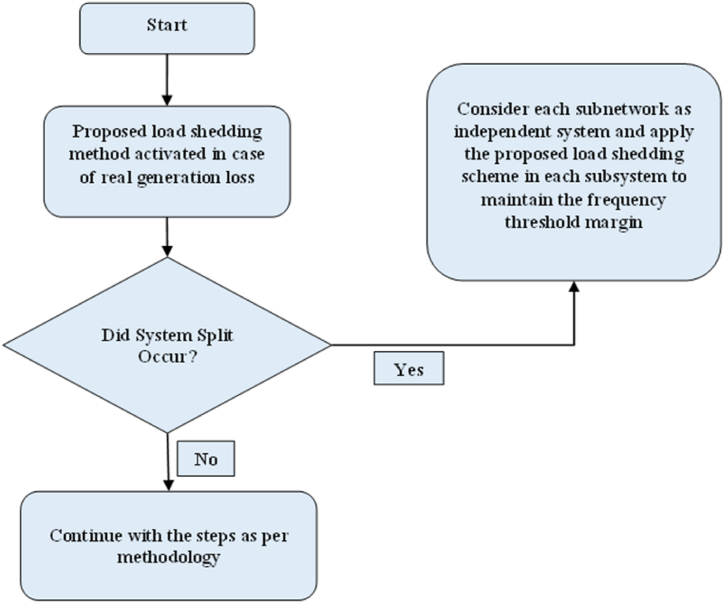
Flowchart of the methodology in case of system splits.

As up-to-date power networks are getting bigger and complex day by day, system splits are becoming quite common. Usually this occurs to protect the machines from thermal outage and safety of the auxiliary systems. If system split occurs then the proposed scheme can be modified in such a way that it can be implemented to each subnetwork to maintain the frequency threshold limit. That means the scheme will treat each subnetwork as individual system and shed the required number of loads. A flowchart is shown in Fig. 12 showing this step. For detection of system splitting, some methods have been proposed in Refs. [43,44]. This can be utilized with the proposed load shedding methodology.

## 4 Performance Evaluation by comparison with another adaptive load shedding scheme

Performances of the proposed strategy are contrasted with those of existing approaches in this section. In this regard a zone based dynamic adaptive load shedding scheme is taken into consideration [45].

### 4.1 Brief overview

**Fig. 13:**
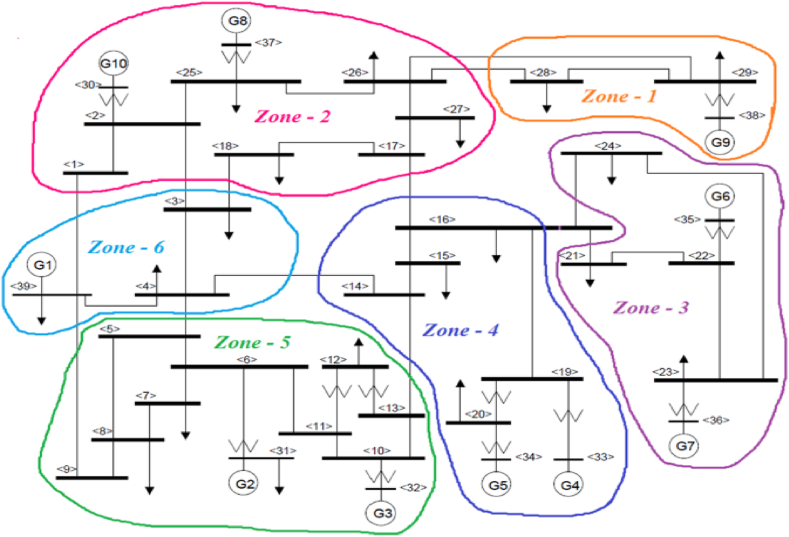
Test system for zone-based load shed method.

In this method the test system has six zones which are on basis of geographical proximity of generators as shown in Fig. 13. It is mentioned here that, in this load shedding there is no feature regarding addition of BESS. So, the only switching event occurring is the feeder breakers switching.

### 4.2 Performance evaluation

The suggested scheme's and the zone-based load shedding scheme's performances are compared for the four cases described in the previous section.

#### 4.2.1 Case-1: 800 MW generation loss with PV injection 250 MW

**Fig. 14:**
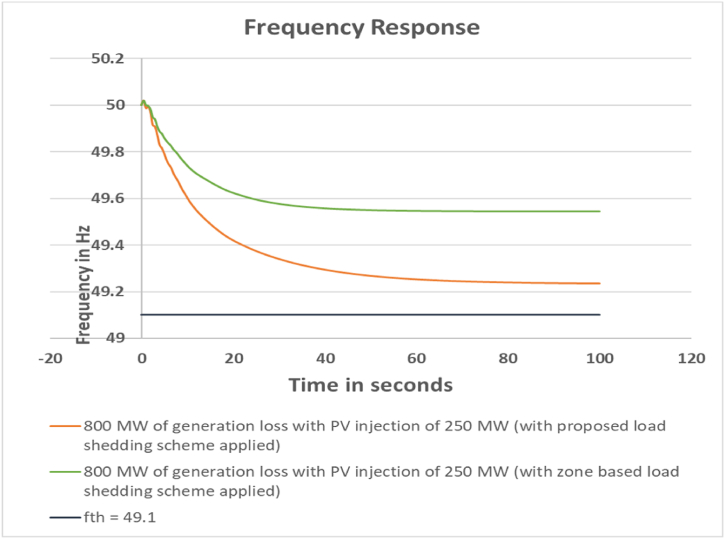
Frequency response comparison between proposed and zone based load shed scheme for Case 1.

For this case, the calculated load shed amount to retain the system frequency within 49.10 Hz by the zone based method is 632 MW. The actual load shed amount is 637 MW. On the other hand, the calculated load shed amount by the proposed scheme for case 1 is 235 MW. It can be observed here that, additional load shed is required for zone based load shedding scheme. The comparison is shown in Table 10. The output frequency response comparison between the proposed and zone based load shedding scheme is depicted in Fig. 14.

**Table 10:** Loadshed Data Comparison for Case 1.

Sl. No.	Bus Number	Calculated Loadshed Amount for proposed scheme (MW)	Calculated Loadshed Amount for zone based scheme (MW)	Actual Loadshed for proposed scheme (MW)	Actual Loadshed for zone based scheme (MW)
1	Bus_28	5	42	0	0
2	Bus_29	6	67	0	110
3	Bus_18	8	20	23	23
4	Bus_25	7	29	0	24
5	Bus_26	5	11	10	11
6	Bus_27	11	50	0	50
7	Bus_16	10	5	20	0
8	Bus_21	18	19	20	30
9	Bus_22	20	18	20	0
10	Bus_23	17	13	15	30
11	Bus_24	13	11	15	0
12	Bus_14	13	56	0	55
13	Bus_15	18	87	30	100
14	Bus_20	14	58	0	50
15	Bus_7	11	28	20	34
16	Bus_8	18	59	20	50
17	Bus_12	9	17	10	25
18	Bus_3	5	0	0	0
19	Bus_4	19	28	20	30
20	Bus_39	8	14	15	15
**Total load shed**		**235**	**632**	**238**	**637**

From the frequency response comparison, it can be observed that the zone based method arrests the system frequency well within the threshold value. But the amount of load shed required is quite higher compared to the proposed scheme. It is almost 3 times higher. Also, there is no provision for BESS in the zone based load shedding method.

#### 4.2.2 Case-2: 800 MW generation loss with PV injection 500 MW

For this case, the calculated load shed amount to arrest the system frequency within 49.10 Hz by the zone based method is 632 MW. The actual load shed amount is 637 MW. On the other hand, the calculated load shed amount by the proposed scheme for case 1 is 235 MW. It can be observed here that, additional load shed is required for zone based load shedding scheme. The comparison is shown in Table 11.

**Table 11:** Loadshed Data Comparison for Case 2

Sl. No.	Bus Number	Calculated Loadshed Amount for proposed scheme (MW)	Calculated Loadshed Amount for zone based scheme (MW)	Actual Loadshed for proposed scheme (MW)	Actual Loadshed for zone based scheme (MW)
1	Bus_28	5	42	0	0
2	Bus_29	6	67	0	110
3	Bus_18	8	20	23	23
4	Bus_25	7	29	0	24
5	Bus_26	5	11	10	11
6	Bus_27	11	50	0	50
7	Bus_16	10	5	20	0
8	Bus_21	18	19	20	30
9	Bus_22	20	18	20	0
10	Bus_23	17	13	15	30
11	Bus_24	13	11	15	0
12	Bus_14	13	56	0	55
13	Bus_15	18	87	30	100
14	Bus_20	14	58	0	50
15	Bus_7	11	28	20	34
16	Bus_8	18	59	20	50
17	Bus_12	9	17	10	25
18	Bus_3	5	0	0	0
19	Bus_4	19	28	20	30
20	Bus_39	8	14	15	15
**Total load shed**		**235**	**632**	**238**	**637**

**Fig. 15:**
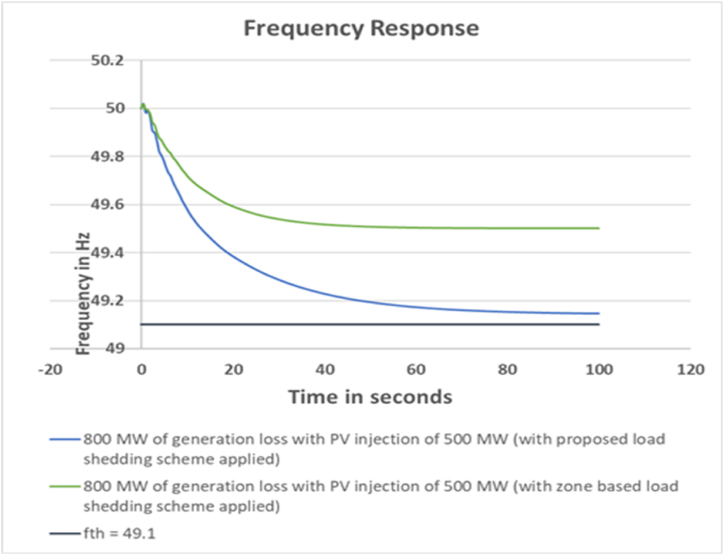
Frequency response comparison between proposed and zone based load shed scheme for Case 2.

The output frequency response comparison between the proposed and zone based load shedding scheme is depicted in Fig. 15.

From the frequency response comparison, it can be concluded that, zone based method arrests the system frequency well within the threshold value in case of any generation loss events. But the amount of load shed required is quite higher compared to the proposed scheme. It is almost 3 times higher. Also, there is no provision for BESS in the zone based load shedding method. Additionally, it can also be observed that due to injection of more renewable generation, the output frequency response has slightly deteriorated compared to case 1. This occurred due to lack of inertia in the system.

#### 4.2.3 Case-3: 1000 MW generation loss with PV injection 500 MW

**Fig. 16:**
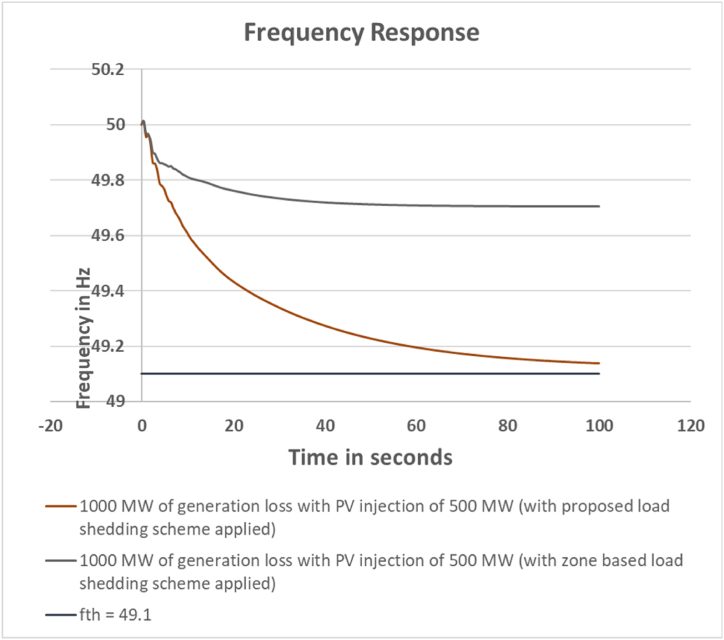
Frequency response comparison between proposed and zone based load shed scheme for Case 3.

For this case, the generation loss amount has been increased to 1000 MW. Consequently, more feeders need to be cut to keep the system frequency within acceptable range. Hence, the calculated load shed amount to arrest the system frequency within 49.10 Hz by the zone based method is 832 MW. The actual load shed amount is 834 MW. On the other hand, the calculated load shed amount by the proposed scheme for this case is 399 MW. It can be observed here that, additional load shed is required for zone based load shedding scheme. The comparison is shown in Table 12. The output frequency response comparison between the proposed and zone based load shedding scheme is depicted in Fig. 16.

**Table 12:** Loadshed Data Comparison for Case 3.

Sl. No.	Bus Number	Calculated Loadshed Amount for proposed scheme (MW)	Calculated Loadshed Amount for zone based scheme (MW)	Actual Loadshed for proposed scheme (MW)	Actual Loadshed for zone based scheme (MW)
1	Bus_28	9	55	10	60
2	Bus_29	11	88	0	80
3	Bus_18	13	26	23	30
4	Bus_25	11	38	0	40
5	Bus_26	9	14	20	11
6	Bus_27	18	65	21	70
7	Bus_16	16	7	0	0
8	Bus_21	31	25	0	30
9	Bus_22	35	24	70	20
10	Bus_23	29	17	30	20
11	Bus_24	22	14	30	15
12	Bus_14	22	74	25	0
13	Bus_15	31	115	30	170
14	Bus_20	24	76	20	0
15	Bus_7	19	37	20	125
16	Bus_8	30	78	30	0
17	Bus_12	15	23	15	66
18	Bus_3	8	1	12	42
19	Bus_4	32	37	30	30
20	Bus_39	14	18	15	25
**Total load shed**		**399**	**832**	**401**	**834**

In the third case, it can be observed that the load shed amount for zone based scheme is almost twice than the proposed scheme. The proposed scheme has successfully kept the frequency within acceptable limits by shedding less load.

#### 4.2.4 Case-4: 1000 MW generation loss with PV injection 1500 MW

**Fig. 17:**
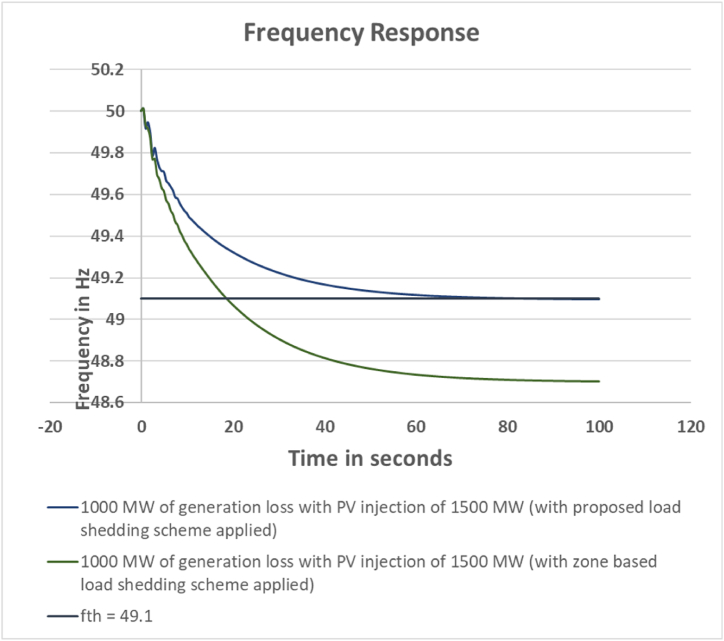
Frequency response comparison between proposed and zone based load shed scheme for Case 4.

The fourth case is considered as the extreme case of contingency where there is injection of 1500 MW renewable generation in place of conventional energy sources. The system inertia has been reduced significantly. The generation loss event considered to be 1000 MW. As before, the zone based method has calculated the load shed amount to be 832 MW for arresting the frequency within threshold limit. The actual load shed done in the system is 834 MW. Whereas, the proposed scheme has calculated the load shed amount to be 652 MW to keep the frequency within threshold value. The comparison is shown in Table 13. The output frequency response comparison between the proposed and zone based load shedding scheme is depicted in Fig. 17.

**Table 13:** Loadshed Data Comparison for Case 4.

Sl. No.	Bus Number	Calculated Loadshed Amount for proposed scheme (MW)	Calculated Loadshed Amount for zone based scheme (MW)	Actual Loadshed for proposed scheme (MW)	Actual Loadshed for zone based scheme (MW)
1	Bus_28	15	55	16	60
2	Bus_29	17	88	0	80
3	Bus_18	21	26	30	30
4	Bus_25	18	38	24	40
5	Bus_26	15	14	15	11
6	Bus_27	29	65	30	70
7	Bus_16	27	7	29	0
8	Bus_21	50	25	50	30
9	Bus_22	56	24	50	20
10	Bus_23	47	17	50	20
11	Bus_24	36	14	30	15
12	Bus_14	36	74	30	0
13	Bus_15	51	115	45	170
14	Bus_20	40	76	0	0
15	Bus_7	31	37	0	125
16	Bus_8	50	78	130	0
17	Bus_12	25	23	22	66
18	Bus_3	13	1	12	42
19	Bus_4	54	37	50	30
20	Bus_39	21	18	25	25
**Total load shed**		**652**	**832**	**638**	**834**

In the fourth case, it is obvious from the output frequency response graph that the zone based method has failed to maintain the threshold frequency. This is because as there is large injection of PV in this case the system inertia has been reduced greatly. As a result, the scheme only calculated the load shed amount based on the generation loss amount (1000 MW) which is equal to third case. It neglects the effect of green energy resources. In order to keep the frequency above 49.10 Hz, additional load shed will be required for the zone based load shed method. On the other hand, the proposed scheme has successfully kept the system frequency within the limit 49.10 Hz with load shed amount of 638 MW.

## 5 Conclusion

This research work focuses on developing an adaptive load shed technique for renewable integrated power network that considers the generation loss amount, injection of renewable energy resources, inclusion of BESS for frequency arresting, impact of voltage and frequency stability indices for determining highly perturbed bus in case of a contingency event, frequency response characteristic etc. of a system. The scheme results in an adequate amount of load shedding scheme with dynamic correction for feeders located in different bus. The suggested scheme has been applied to a modified version of 39 bus New England test system.

There are four scenarios considering different combination of the amount of injection of PV based power generation sources with conventional generation loss of 800 MW and 1000 MW. The threshold frequency is considered 49.10 Hz. The total amount of BESS is 300 MW. The designed load shed scheme has successfully maintained the frequency threshold level 49.10 Hz for all scenario presented with a minimal amount of load shedding. Hence, it can be stated that the proposed methodology effectively maintains frequency stability for a modern power system with large-scale PV generation through adaptive feeder selection for load shed.

The proposed load shed method has been compared with another zone based adaptive scheme [45]. It has been found that the designed method provides satisfactory frequency response with lesser load shed. In addition, the proposed scheme has better result in case of PV injection compared to zone based scheme.

Last but not the least, a sudden increase in load cannot be attributed to the activation of the suggested procedure. The suggested method, however, can be easily expanded to begin load shedding following such an incident. Also, the scenario with loss of a renewable generation or change of PV injection has not been addressed which can also be large scope of future works for the suggested methodology.

## CRediT authorship contribution statement

**Sk Fahim Abrar:** Writing – original draft, Software, Resources, Methodology, Investigation, Formal analysis, Data curation, Conceptualization. **Nahid-Al Masood:** Writing – review & editing, Supervision, Conceptualization. **Mohammad Jahangir Alam:** Writing – review & editing, Supervision.

## Data availability statement

The data associated with the study have not been deposited into a publicly available repository. The data that have been used is confidential.

## Declaration of competing interest

The authors declare that they have no known competing financial interests or personal relationships that could have appeared to influence the work reported in this paper.
